# Very pronounced bowel sparing during radiation therapy for anal carcinoma using a natural spacer (Myoma) – a case report

**DOI:** 10.1186/s13014-024-02530-6

**Published:** 2024-10-15

**Authors:** L. Hoeng, A. K. Exeli, G. A. Krombach, T. Schwandner, L. Agolli, D. Habermehl

**Affiliations:** 1 Department of Radiation Oncology, Justus-Liebig-University Giessen, Giessen-Marburg University Hospital, Giessen, Germany; 2 Department of Diagnostic and Interventional Radiology, Justus-Liebig-University Giessen, Giessen-Marburg University Hospital, Giessen, Germany; 3 Department of General and Visceral Surgery, Asklepios Hospital Lich, Lich, Germany; 4 Medical Physics, Department of Radiation Oncology, Giessen-Marburg University Hospital, Giessen, Germany

**Keywords:** Anal carcinoma, Bowel-sparing radiotherapy, Myomatous uterus, Myoma, Toxicity, Abdominal spacer

## Abstract

**Background:**

Using dose-painted intensity-modulated radiation therapy, specific dose volume constraints or implantation of tissue expanders prior to radiotherapy are validated options for reducing radiation dose on the bowel and therefore minimizing acute gastrointestinal toxicity during chemoradiation for anorectal malignancies. We describe the rare case of a female patient with a locally advanced anal carcinoma where a large myomatous uterus served as a natural spacer to protect the bowel during radiation therapy.

**Case presentation:**

Initially the patient presented with anal pain, proctoscopy followed by an excisional biopsy confirmed the diagnosis of a squamous cell carcinoma of the anus. Imaging examination showed a locally advanced tumor and in addition a large uterus with typical leiomyomas up to 11.5 cm in diameter. The patient underwent chemoradiation; because of the large leiomyomas there was almost no dose burden for the small intestine and therefore practically no gastrointestinal toxicity.

**Conclusion:**

As we know, this report describes the situation that a large myomatous uterus served as a natural spacer during radiation therapy in a way that is unique to date.

## Background

A significant challenge during combined chemoradiation (CRT) for anal carcinoma is minimizing radiation-induced toxicity to surrounding organs, particularly the small and large bowel. Previous studies showed the effectiveness of dose-painted intensity-modulated radiation therapy (IMRT) in reducing acute gastrointestinal toxicity during CRT for anorectal malignancies [1–4]. It was also noted that specific dose-volume constraints may help to mitigate bowel toxicity [5]. In addition, some groups suggested the implantation of a tissue expander prior to radiation therapy (RT) to keep the bowel away from the target volume with the aim to reduce the risk of acute and late gastrointestinal toxicity [6]. Nevertheless, bowel exposure and acute and late bowel toxicity remains a challenge. This case report describes the dose-reducing effect of a naturally occurring large myomatous uterus, serving as a natural spacer.

## Case presentation

**Fig. 1:**
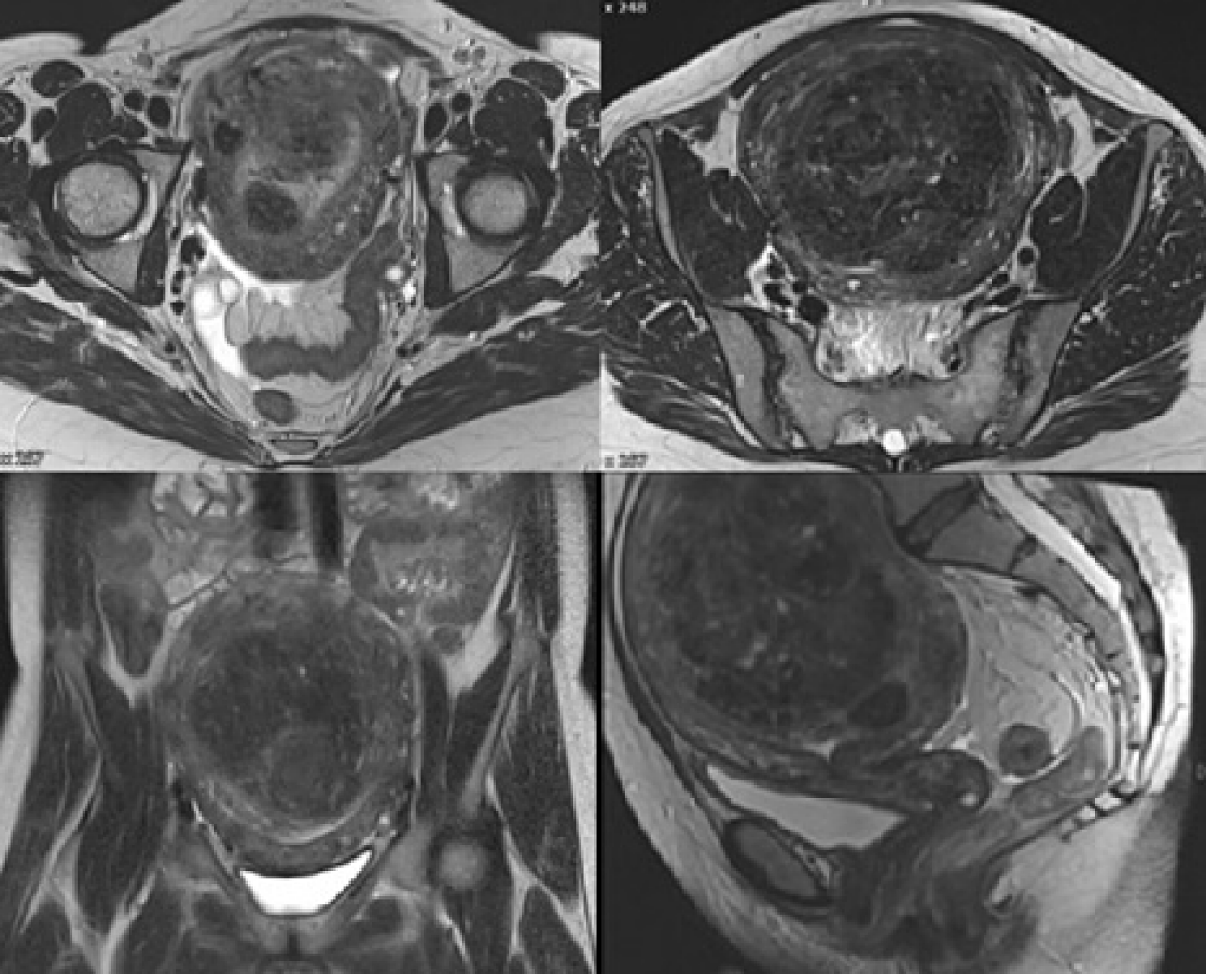
MRI with large myomatous uterus

We report a case of a 49-year-old female patient, who presented with anal pain. Proctoscopy showed an ulcerated lesion, an excisional biopsy confirmed the diagnosis of a squamous cell carcinoma of the anus. MRI and CT scans showed a locally advanced tumor without distant metastasis. Considering imaging and histopathologic results, tumor stage was pT2 cN0 cM0 G3 L0 V0 R1. Additionally, the images showed a large uterus with typical leiomyomas up to 11.5 cm in diameter (Fig. 1). These uterine leiomyomas were previously diagnosed by her gynecologist; hysterectomy was planned after CRT and confirmed complete remission of the anal carcinoma.

### Intervention

The patient was treated with a total dose of 45 Gy in 25 fractions to elective nodal regions, including pelvic lymph nodes and 50 Gy to the primary tumour region as simultaneous integrated boost (SIB), delivered as volumetric modulated arc therapy (VMAT). The patient received 5-fluorouracil (5-FU) and mitomycin C concurrently with RT. Planning was based on CT and MRI scans. The myomatous uterus was contoured also based on the MRI imaging and was utilized as a natural spacer to increase the distance between the radiation fields and the bowel. Verification of positioning during RT was performed with image-guidance (IGRT) using daily cone-beam computed tomography (CBCT).

### Dosimetric benefits

**Fig. 2:**
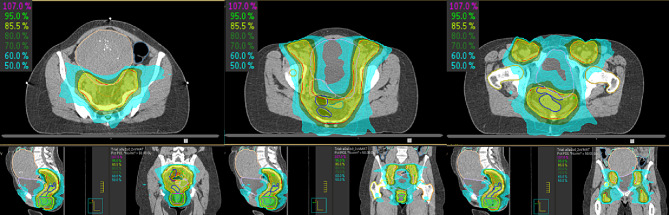
Dose distribution in three representative slices (axial, coronal, sagital) for SIB (purple), PTV (red), CTV (orange), myoma (skin), bladder (lavender), colon (steelblue), femoral heads (yellow, orange), sigmoid colon (teal) and small bowel (aquamarine) with isodose lines (50% light blue, 60% light blue, 70% dark green, 80% dark green, 85.5% yellowgreen, 95% green, 107% purple)

**Fig. 3:**
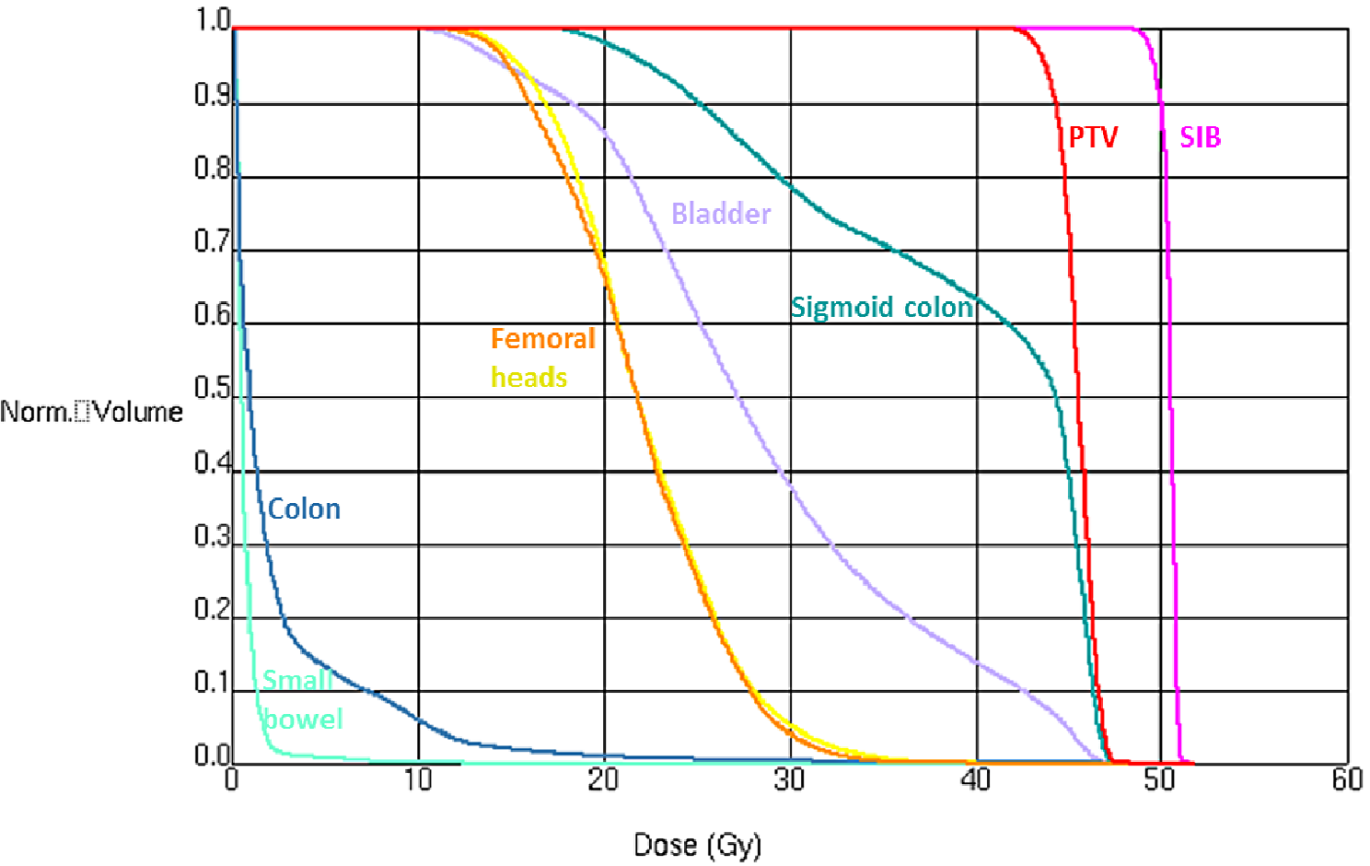
Dose-volume histogram (DVH) of the investigated case for small bowel (aquamarine), colon (steelblue), femoral heads (left – orange, right – yellow), bladder (lavender), sigmoid colon (teal), PTV (red) and SIB (purple). X-axis shows the absolute dose in Gray (Gy). Y-axis shows the relative volume of the respective ROI in cubic centimeters (cc).

The presence of the myoma significantly reduced the radiation dose to the bowel – especially to the small bowel, as confirmed by dosimetric analysis (Fig. 2). Standard dose constraints for the small bowel typically aim to keep the volume of bowel receiving higher doses below a certain threshold (e.g. D_max_ 54 Gy and V45 < 150 cc for normofractionated RT). In this case, the Dose-volume-histogram (DVH) showed a marked reduction in the volume of small bowel receiving high-dose. The small bowel received a dose maximum of only 12.5 Gy, V45 = 8.2 cc and a mean dose of 0.7 Gy (Fig. 3).

The most important dose parameters are summarized in Table 1, including dose minimum (D_min_), dose maximum (D_max_) and mean dose (D_mean_).

**Table 1:**
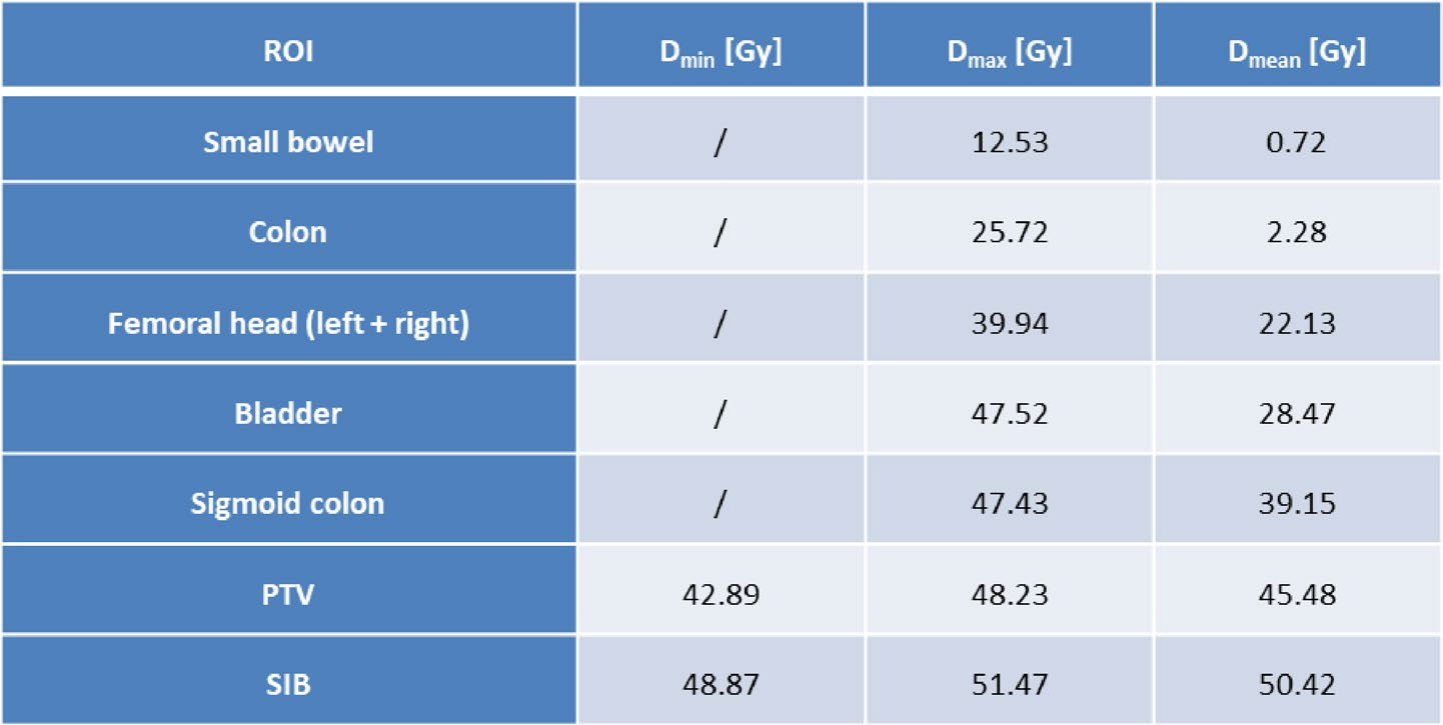
Dose parameters in Gy for the in Fig. 3 shown region of interests

### Treatment tolerance

The treatment was well tolerated. The patient developed manageable side effects, including bladder irritation and skin erythema. Correlating to the dosimetric benefits, the patient described no gastrointestinal side effects except of moderate rectal pain.

## Discussion

Despite technological advances in radiotherapy treatment planning including intensity-modulated RT, volumetric-modulated RT, IGRT, online and offline adaptive RT approaches and charged particle therapy with protons and carbon ions, gastrointestinal toxicity remains a therapy-limiting issue for many indications [7–10]. In the past, some institutions successfully tried to overcome the gastrointestinal dose deposition in abdominal and pelvic radiotherapy with the surgical implantation of prosthetic, silastic, saline-filled tissue expanders [11–14]. A recent retrospective analysis on 29 children that received pelvic or abdominal RT after implantation of a silicone tissue-expander prosthesis (STEP) reports a reduced bowel dose over 40 Gy by 64% [12]. The 15-year complication-free survival of the irradiated long-term surviving children was 70%. However, the implantation itself represents an additional surgical procedure with foreign material with the potential of causing postoperative complications, repeat operations and a relevant delay of the planned RT treatment [6]. It is certainly very rare that an anatomical anomaly can lead to a significantly better dose distribution, especially in patients who are undergoing radiotherapy for anal carcinoma, which is more likely to have side effects.

Taking our case as a starting point, various anatomical structures or abnormalities, if present, can be used as natural protectors in the case of high-dose radiotherapy. The use of appropriate diagnostic imaging can help in identifying and defining these structures or abnormalities.

## Conclusion

Our report illustrates the unique opportunity of a large myomatous uterus to serve as a natural spacer to protect the bowel during RT for anal carcinoma. To our knowledge this report describes the rare situation that an anatomic/pathological anomaly exceptionally leads to a very advantageous dose distribution with almost no dose burden of the small intestine.

## Abbreviations

5-FU: 5-Fluorouracil
CBCT: Cone-Beam Computed Tomography
CC: Cubic Centimeters
CRT: Chemoradiotherap
CT: Computed Tomography
CTV: Clinical Target Volume
DVH: Dose-Volume-Histogram
Gy: Gray
IGRT: Image-Guided Radiotherapy
IMRT: Intensity-Modulated Radiation Therapy
MRI: Magnetic Resonance Imaging
PTV: Planning Target Volume
ROI: Region Of Interest
RT: Radiation Therapy
SIB: Simultaneous Integrated Boost
VMAT: Volumetric Modulated Arc Therapy

## References

[1] Dapper H, Oechsner M, Hirche C, Münch S, Sauter C, Borm K, Peeken JC, Combs SE, Habermehl D. Dosimetric comparison of different radiation techniques (IMRT vs. 3-dimensional) of the true (deep) ano-inguinal lymphatic drainage of anal cancer patients. Radiat Oncol. 2018;13(1):227. 10.1186/s13014-018-1174-z. 30466454 10.1186/s13014-018-1174-z PMC6249729

[2] Dapper H, Oechsner M, Münch S, Borm K, Peeken J, Mayinger M, Combs SE, Habermehl D. Dosimetric analysis and comparison of reduced longitudinal cranial margins of VMAT-IMRT of rectal cancer. Radiat Oncol. 2018;13(1):169. 10.1186/s13014-018-1120-0. 30189877 10.1186/s13014-018-1120-0 PMC6127934

[3] Dapper H, Rodríguez I, Münch S, Peeken JC, Borm K, Combs SE, Habermehl D. Impact of VMAT-IMRT compared to 3D conformal radiotherapy on anal sphincter dose distribution in neoadjuvant chemoradiation of rectal cancer. Radiat Oncol. 2018;13(1):237. 10.1186/s13014-018-1187-7. 30509284 10.1186/s13014-018-1187-7 PMC6276230

[4] Regnier A, Ulbrich J, Münch S, Oechsner M, Wilhelm D, Combs SE, Habermehl D. Comparative Analysis of Efficacy, toxicity, and patient-reported outcomes in rectal Cancer patients undergoing preoperative 3D conformal radiotherapy or VMAT. Front Oncol. 2017;7:225. 10.3389/fonc.2017.00225. 28979889 10.3389/fonc.2017.00225 PMC5611394

[5] Olsen JR, Moughan J, Myerson R, Abitbol A, Doncals DE, Johnson D, Schefter TE, Chen Y, Fisher B, Michalski J, Narayan S, Chang A, Crane CH, Kachnic L. Predictors of Radiation Therapy-related gastrointestinal toxicity from Anal Cancer dose-painted intensity modulated Radiation Therapy: secondary analysis of NRG Oncology RTOG 0529. Int J Radiat Oncol Biol Phys. 2017;98(2):400–8. 10.1016/j.ijrobp.2017.02.005. 28463160 10.1016/j.ijrobp.2017.02.005 PMC5639877

[6] Ollivier L, Guilloit JM, Dos Santos M, Guillemette L, Florescu C, M’vondo CM, Meyer E, Galais MP, Corbinais S, Parzy A, Varatharajah S, Lesueur P. Implantation of tissue expander prior to irradiation in the era of intensity modulated radiotherapy: impact on the management of patients with pelvic digestive cancers. Int J Colorectal Dis. 2020;35(3):559–64. 10.1007/s00384-019-03475-z. 31853620 10.1007/s00384-019-03475-z

[7] Guido A, Cuicchi D, Castellucci P, Cellini F, Di Fabio F, Llimpe FLR, Strigari L, Buwenge M, Cilla S, Deodato F, Macchia G, Galietta E, Golfieri R, Ardizzoni A, Zagari RM, Fanti S, Poggioli G, Fuccio L, Morganti AG. Adaptive individualized high-dose preoperAtive (AIDA) chemoradiation in high-risk rectal cancer: a phase II trial. Eur J Nucl Med Mol Imaging. 2023;50(2):572–80. 10.1007/s00259-022-05944-0. 36127416 10.1007/s00259-022-05944-0 PMC9816267

[8] Habermehl D, Wagner M, Ellerbrock M, Büchler MW, Jäkel O, Debus J, Combs SE. Reirradiation using Carbon ions in patients with locally recurrent rectal Cancer at HIT: first results. Ann Surg Oncol. 2015;22(6):2068–74. 10.1245/s10434-014-4219-z. 25384705 10.1245/s10434-014-4219-z

[9] Leung E, Gladwish AP, Davidson M, Taggar A, Velker V, Barnes E, Mendez L, Donovan E, Gien LT, Covens A, Vicus D, Kupets R, MacKay H, Han K, Cheung P, Zhang L, Loblaw A, D’Souza DP. Quality-of-life outcomes and toxic effects among patients with cancers of the Uterus treated with stereotactic pelvic adjuvant Radiation Therapy: the SPARTACUS Phase 1/2 Nonrandomized Controlled Trial. JAMA Oncol. 2022;8(6):1–9. 10.1001/jamaoncol.2022.0362. 35420695 10.1001/jamaoncol.2022.0362 PMC9011178

[10] Mendez LC, Arifin AJ, Bauman GS, Velker VM, Ahmad B, Lock M, Venkatesan VM, Sexton TL, Rodrigues GB, Chen J, Schaly B, Warner A, D’Souza DP. Is hypofractionated whole pelvis radiotherapy (WPRT) as well tolerated as conventionally fractionated WPRT in prostate cancer patients? The HOPE trial. BMC Cancer. 2020;20(1):978. 10.1186/s12885-020-07490-0. 33036579 10.1186/s12885-020-07490-0 PMC7547418

[11] Hong A, Stevens G, Stephen M. Protection of the small bowel during abdominal radiation therapy with a tissue expander prosthesis. Aust N Z J Surg. 2000;70(9):690–2. 10.1046/j.1440-1622.2000.01911.x. 10976905 10.1046/j.1440-1622.2000.01911.x

[12] Missohou F. Use of expanders for bowel protection in pediatric pelvic tumor radiation therapy: 15 years of tolerance results. Pract Radiat Oncol. 2018 Jul-Aug;8(4):e224–30. 10.1016/j.prro.2017.12.002. 10.1016/j.prro.2017.12.002 29452875

[13] Pérez-Muñoz I, Grimer RJ, Spooner D, Carter S, Tillman R, Abudu A, Jeys L. Use of tissue expander in pelvic Ewing’s sarcoma treated with radiotherapy. Eur J Surg Oncol. 2014;40(2):197–201. 10.1016/j.ejso.2013.09.001. 24084085 10.1016/j.ejso.2013.09.001

[14] Ravn S, Pearcey R, Capstick V. Use of a tissue expander to protect small bowel during radiotherapy in a cervical cancer patient with severe Crohn’s disease. Gynecol Oncol Rep. 2015;14:16–9. 10.1016/j.gore.2015.08.004. 26793765 10.1016/j.gore.2015.08.004 PMC4688864

